# Unveiling the fructose metabolism system in *Staphylococcus aureus*: insights into the regulatory role of FruR and the FruRKT operon in bacterial fitness

**DOI:** 10.1186/s12866-023-03151-x

**Published:** 2024-01-04

**Authors:** Yan Ge, Daiyu Li, Ning Wang, Yun Shi, Gang Guo, Liyuan Fang, Quanming Zou, Qiang Liu

**Affiliations:** 1 West China Biopharmaceutical Research Institute, West China Hospital, Sichuan University, Chengdu, China; 2 Genomics Center of Core Facilities, West China Hospital, Sichuan University, Chengdu, China; 3 National Engineering Research Center of Immunological Products, Department of Microbiology and Biochemical Pharmacy, College of Pharmacy, Third Military Medical University, Chongqing, China

**Keywords:** *S. Aureus*, Transcriptional regulator, DeoR, Fructose metabolism

## Abstract

**Background:**

The utilization of fructose as a carbon source and energy provider plays a crucial role in bacterial metabolism. Additionally, fructose metabolism directly impacts the pathogenicity and virulence of certain pathogenic microorganisms.

**Results:**

In this study, we report the discovery of a fructose phosphotransferase system (PTS) in *S. aureus*. This system comprises three genes, namely *fruR*, *fruK*, and *fruT*, which are co-located in an operon that is indispensable for fructose utilization in *S. aureus*. Our findings confirm that these three genes are transcribed from a single promoter located upstream of the *fruRKT* operon. The *fruR* gene encodes a DeoR-type transcriptional regulator, designated as FruR, which represses the expression of the *fruRKT* operon by direct binding to its promoter region. Significantly, our experimental data demonstrate that the *fruRKT* operon can be induced by fructose, suggesting a potential regulatory mechanism involving intracellular fructose-1-phosphate as a direct inducer. Furthermore, we conducted RNA-seq analysis to investigate the specificity of FruR regulation in *S. aureus*, revealing that the *fruRKT* operon is predominantly regulated by FruR.

**Conclusions:**

In summary, this study has uncovered a fructose phosphotransferase system (PTS) in *S. aureus*, highlighting the essential role of the *fruR*, *fruK*, and *fruT* genes in fructose utilization. We confirmed their co-location within an operon and established FruR as a key regulator by binding to the operon’s promoter. Importantly, we demonstrated that fructose can induce this operon, possibly through intracellular fructose-1-phosphate. Our identification of this PTS system represents the initial characterization of a fructose metabolism system in *S. aureus*.

**Supplementary Information:**

The online version contains supplementary material available at 10.1186/s12866-023-03151-x.

## Background

Bacterial carbohydrate utilization systems play a crucial role in acquiring carbon sources and energy. While extensive research has been conducted on the utilization systems of glucose and lactose, other sugar utilization systems, especially the metabolism and regulation of fructose, have received relatively less attention. Fructose utilization in bacteria involves three main pathways [1]. The primary mechanism for fructose utilization in bacteria is through sugar-specific phosphorylation transport systems (PTS), although alternative non-PTS uptake systems have also been documented [2]. Within the PTS system, fructose is transported into the bacterial cell by a membrane-associated diphosphoryl transfer protein (FruT). Concurrently, fructose is phosphorylated to fructose-1-phosphate through the transfer of a phosphate group from phosphoenolpyruvate to fructose. Subsequently, the generated fructose-1-phosphate is further phosphorylated to fructose-1,6-bisphosphate by ATP and FruK (1). The *fruRKT* operon in bacteria governs the expression of proteins involved in fructose utilization. The transcriptional regulation of this operon is primarily orchestrated by a transcriptional regulator known as FruR, which is encoded within the operon itself [3–5].

Various fructose (*fru*) operons encoding enzymes have been identified in different bacterial groups, including *Corynebacterium glutamicum*, *Spiroplasma citri*, *Streptococcus mutans*, and *Streptococcus gordonii* [3–6]. In *S. citri*, the *fru* operon has been shown to play a role in fructose metabolism and pathogenesis [7]. In oral streptococci such as *S. mutans* and *S. gordonii*, the presence of high-affinity sugar utilization systems like the PTS Fru contributes to their survival between meals and aids in the production of acids that contribute to tooth decay. Furthermore, the *fru* operon has been implicated in biofilm formation by *S. gordonii*, promoting the accumulation and persistence of bacteria on oral surfaces [6]. Despite the conservation of genetic organization of fructose utilization operons across various genera, such as *Staphylococcus* [5], the specific role of these operons in fructose metabolism in the important pathogen *Staphylococcus aureus* has not been experimentally studied.

An intriguing aspect of the fructose utilization genes in these bacteria is the presence of a regulatory gene from the DeoR family located upstream. Additionally, all three genes, including the regulatory gene, are transcribed as a single unit from a shared promoter region. The regulation of the fructose utilization operon has been studied in two bacterial species, *S. citri* and *S. gordonii*. In both species, the presence of fructose in the culture medium was found to enhance the transcription of these operons. Interestingly, the regulatory role of FruR in *S. citri* and *S. gordonii* is quite remarkable. In *S. citri*, FruR acts as an activator, promoting transcription of the *fru* operon when fructose is present [3]. However, in *S. gordonii*, FruR acts as a repressor, inhibiting *fru* operon transcription [6]. This findings indicated the diversity in the molecular mechanisms underlying the regulation of fructose metabolism by FruR in the PTS.

In this study, we present the initial characterization of the *fru* operon (*fruRKT*) responsible for fructose metabolism in *S. aureus*. The primary objective of our investigation was to elucidate the functional aspects and regulatory mechanisms governing the fructose utilization pathway mediated by the *fruRKT* operon in *S. aureus*.

## Results

### Gene ***sa03665*** encodes a DeoR-type transcriptional regulator in a sugar metabolism operon

**Fig. 1:**
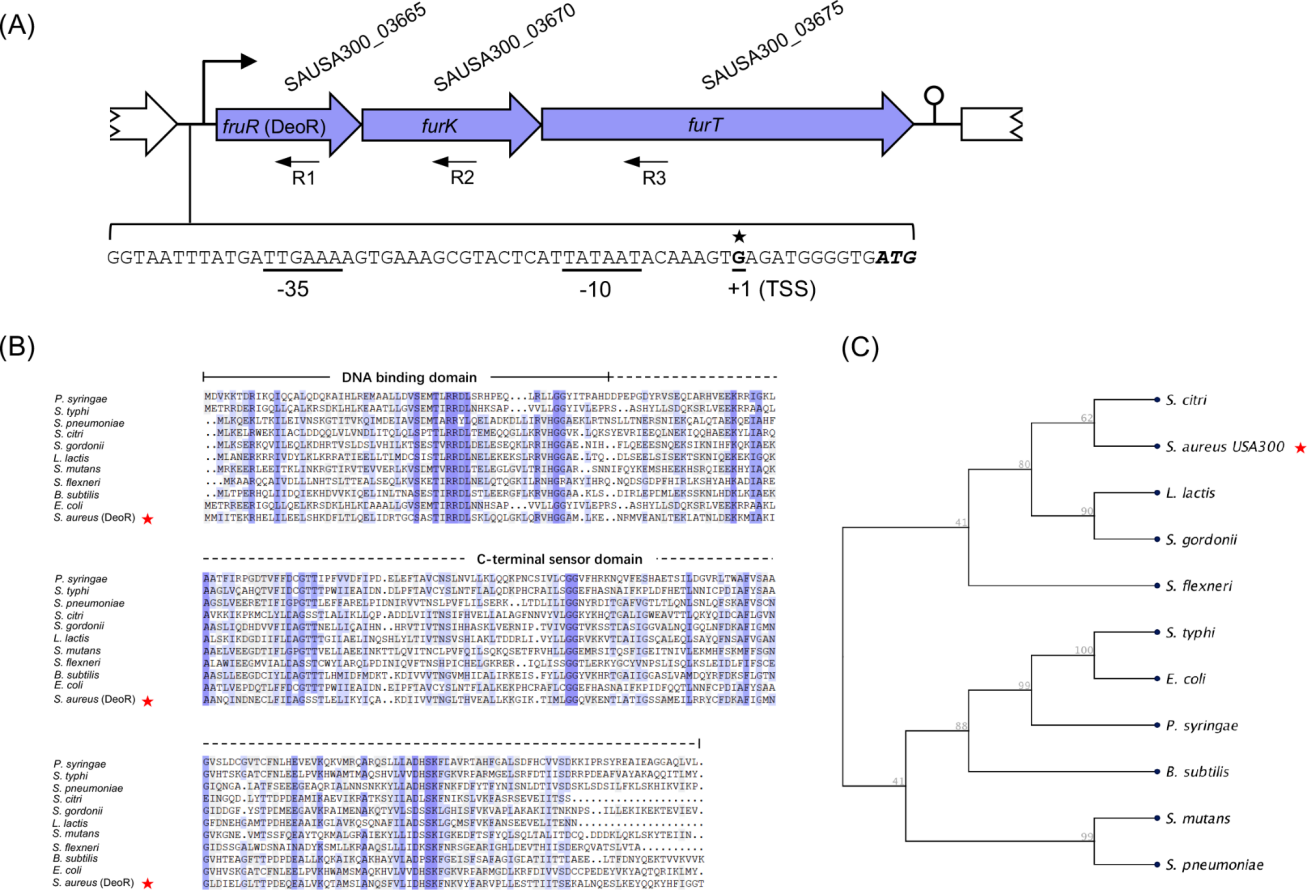
(**A**) Genetic organization of *fruRKT* operon. The *fruRKT* genes were transcribed from the identical site “G”, marked with a star, which corresponds to the “+1” position. A prototypical σ^70^-regulated promoter featuring the conserved − 10 and − 35 regions was indicated. (**B**) Sequence alignment of FruR protein from *S. aureus* (indicated by a red star) and established DeoR proteins from diverse bacterial species, with the respective organism names provided in the materials and methods section. (**C**) The phylogenetic tree derived from the alignment depicted in figure B was constructed utilizing the neighbor-joining method and the Jukes-Cantor protein distance model to compute the resultant tree

In the genome of *S. aureus* USA300_FPR3757, genes with locus tag SAUSA300_03665 (*sa03665*), SAUSA300_03670 (*sa03670*), and SAUSA300_03675 (*sa03675*) were predicted to be co-expressed in a single operon based on the STRING database prediction (Fig. 1A). The genome annotation indicated that *sa03665* encoded a DeoR/GlpR family transcriptional regulator, while the *sa03670* and *sa03675* encoded 1-phosphofructokinase and fructose transporter, respectively, leading to their designation as *fruK* and *fruT* genes (Fig. 1A).

To verify the membership of SA03665 protein in the DeoR family, a protein sequence alignment was carried out between SA03665 and confirmed DeoR transcription factors. The findings indicated that the SA03665 protein exhibited analogous and conserved amino acid sequences to other known DeoR transcription factors, characterized by the presence of an helix-turn-helix (HTH) DNA-binding domain at the N-terminus and a sensor domain at the C-terminus (Fig. 1B).

A phylogenetic tree was generated based on the alignment, unequivocally indicating that SA03665 shares a close relationship with DeoR protein identified in *S. citri* (Fig. 1C). The genomic context of the DeoR coding gene observed in *S. citri* mirrors that of *S. aureus*, showing that the DeoR coding gene is located upstream of two genes *fruA* and *fruK* [3]. Specifically, the DeoR protein encoded by *fruR* gene in *S. citri* acts as a regulator of its downstream genes *fruAK*, involved in fructose utilization. By extension, *sa03665* gene was designated as *fruR*, given its potential involvement in this pathway in *S. aureus*.

### ***FruRKT*** genes are co-expressed within one operon

In order to verify the hypothesis that *fruRKT* genes are co-expressed in a single operon, the transcriptional start site (TSS) of these genes was determined using 5’ rapid amplification of cDNA end (5’-RACE) with primers R1, R2, and R3, which are complementary to *fruR*, *fruK*, and *fruT* (Fig. 1A). Notably, all primers generated the same 5’ end position “G,” which is located 11 bp upstream of the *fruR* start codon (Fig. 1A), indicating that these three ORFs are transcribed from the same promoter upstream of *fruR* gene. Examination of the sequence upstream of the TSS revealed a classical promoter that is recognized by the σ^70^ family RNA polymerase located immediately upstream of the TSS, which contains a perfectly conserved − 10 AT-rich region (TATAAT) and − 35 box (TTGAAA) (Fig. 1A). Furthermore, a rho-independent terminator structure is located downstream of *fruT* (Fig. 1A). These observations indicated that the *fruRKT* genes are expressed collectively within a single operon.

### ***FruK*** and ***fruT*** are essential for fructose utilization

Given the preliminary annotation of the *fruRKT* genes as encoding the DeoR transcription factor, phosphofructokinase, and fructose transporter, respectively, this current investigation aims to elucidate the specific roles that these three genes play in the process of fructose catabolism within *S. aureus*. Thus, we generated single gene deletion mutants of *fruR* (Δ*03665*), *fruK* (*Δ03670*), *fruT* (*Δ03675*), as well as a double deletion mutant of *fruKT* genes (Δ*03670_03675*), and monitored their growth patterns in synthetic minimal medium (SMM) using fructose as the sole carbon source.

**Fig. 2:**
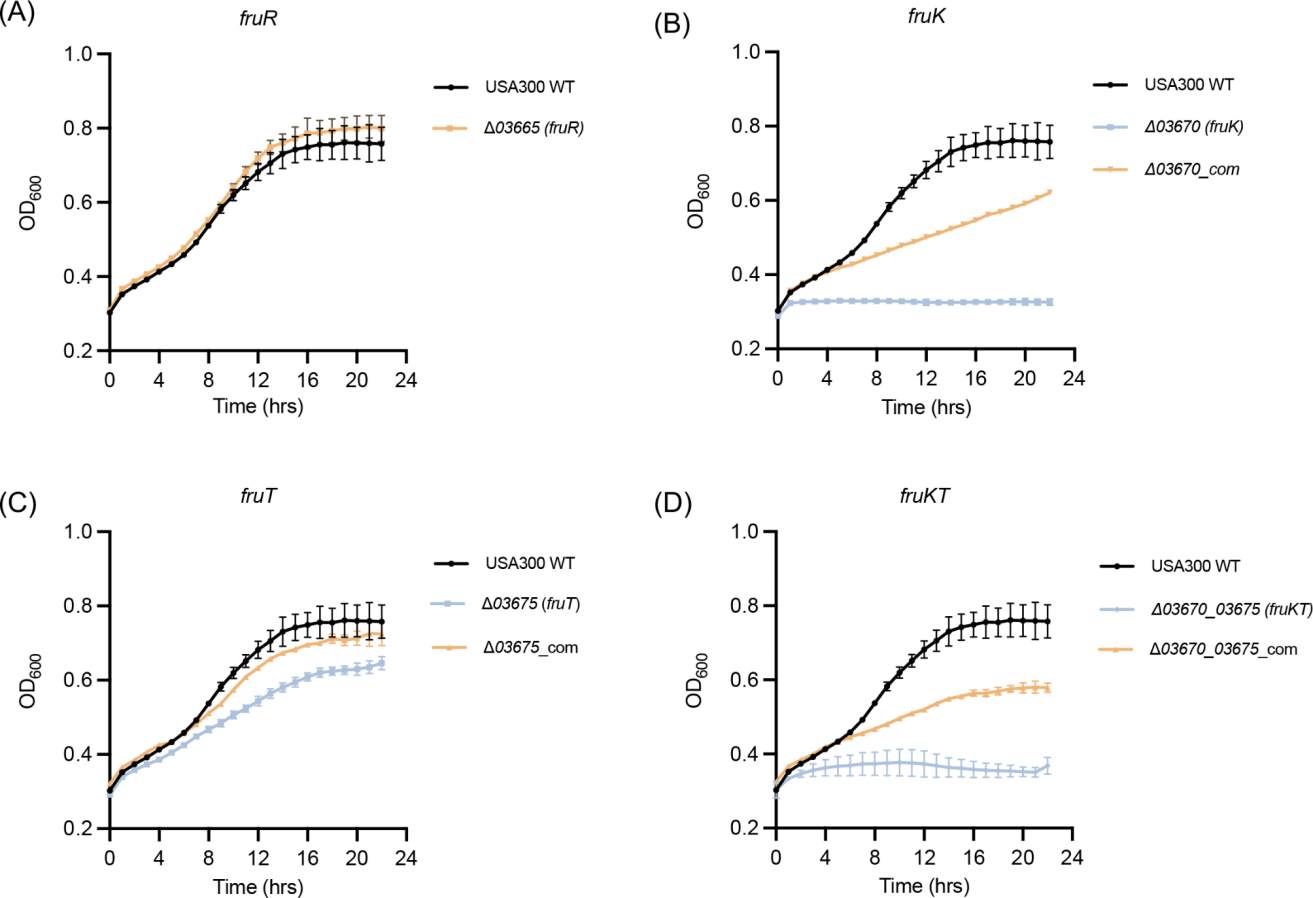
The growth curves of *S. aureus* USA300 WT, as well as gene deletion mutants of *fruR* (Δ*03665*) (A), *fruK* (*Δ03670*) (B), *fruT* (*Δ03675*) (C), and *fruKT* (Δ*03670_03675*) (D), were monitored in the synthetic minimal medium (SMM) supplemented with 0.5% fructose as solo carbon source. Each growth curve was performed three times and the data was presented as the mean plus standard deviation

As demonstrated by the results, *S. aureus* WT were able to thrive in SMM medium supplemented with fructose, indicating that the *S. aureus* can utilize fructose as a carbon source. Interestingly, the growth patterns of Δ*03665* (*fruR*) mutant were similar to those of WT (Fig. 2A), while Δ*03675* (*fruT*) mutant displayed a significant growth deficiency compared to WT (Fig. 2B). Of utmost importance, Δ*03670* (*fruK*) mutant displayed a complete cessation of growth in SMM with fructose as the sole carbon source (Fig. 2C). Remarkably, the phenotype of growth deficiency resulting from the deletion of the *fruT* or *fruK* gene could be restored by the complementary expression of *fruT* or *fruK* gene in their corresponding deletion mutants (Fig. 2B,C). The mutant Δ*03670_03675* (*fruKT*) exhibited the same phenotype as Δ*03670* (*fruK*) (Fig. 2D).

These results revealed that *fruR* gene is dispensable for fructose utilization in *S. aureus*. In contrast, the *fruT* gene plays a critical role in the fructose metabolic pathway in *S. aureus*, as its deletion leads to a compromised, albeit not completely abolished, efficiency of fructose utilization. This suggested that *S. aureus* may have the ability to compensate for this loss by potentially utilizing alternative pathways. Notably, the *fruK* gene is indispensable and serves as a crucial component of the fructose utilization machinery in *S. aureus*.

### FruR functions as a repressor inhibiting the expression of ***fruRKT*** operon

**Fig. 3:**
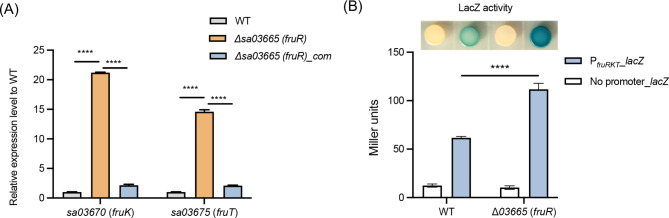
(**A**) RT-qPCR analysis of *fruK* and *fruK* genes expression levels in *S. aureus* USA300 WT, Δ*03665* (*fruR*), and Δ*03665*_com strains cultured in TSB medium. Expression levels were normalized to the WT. (**B**) Beta-galactosidase-based promoter activity assay. *S. aureus* USA300 WT and Δ*03665* (*fruR*) strains carrying the pQLV003 derived P_*fruRKT*__*lacZ* reporter plasmid were grown on TSB X-gal plates at 37 °C for 24 h (up panel). The beta-galactosidase assay of the cell lysates of each strain was quantified, and the strains containing pQLV003 empty vector were used as a control. (^****^P ≤ 0.0001. Each assay was performed three biological replicates and the data was presented as the mean plus standard deviation)

Considering that the *sa03665* (*fruR*) gene encodes a DeoR transcription factor (Fig. 1) that typically regulates its neighboring genes [3, 4], the role of FruR in regulating the expression of *fruK* and *fruT* genes in *S. aureus* was investigated here. To this end, we generated *sa03665* (*fruR*) gene deletion mutant (Δ*03665*) and a complementary strain (Δ*03665*_com), and assessed the expression levels of *fruK* and *fruT* genes in *S. aureus* WT, Δ*03665*, and Δ*03665*_com by RT-qPCR. Of particular note, the expression levels of *fruK* and *fruT* genes in the Δ*03665* mutants were found to increase 15- to 20-fold compared to WT, a phenomenon that was ameliorated upon complementary expression of *sa03665* (*fruR*) in Δ*03665* mutant (Fig. 3A).

To further confirmed the repression role of FruR on the expression of *fruK* and *fruT* genes, a DNA fragment containing the promoter sequence (-105 to + 10) of the *fruRKT* operon was amplified and cloned into a promoter-probe plasmid pQLV003 upstream of the *lacZ* reporter gene. The resultant plasmid was then transformed into both *S. aureus* USA300 WT and Δ*03665* mutant strains, and the promoter activity in each strain was assessed by measuring the beta-galactosidase activity encoded by *lacZ*. The analysis showed that the Δ*03665* mutant, which contained the reporter plasmid, exhibited significantly darker blue colony formation on TSB-X-gal plates and had a 2-fold higher beta-galactosidase activity than the WT strain (Fig. 3B). Collectively, these findings suggest that FruR acts as a transcriptional repressor of the *fruRKT* operon by restraining its promoter activity.

### Fru***R*** binds to the promoter region of Fru***RKT*** operon

**Fig. 4:**
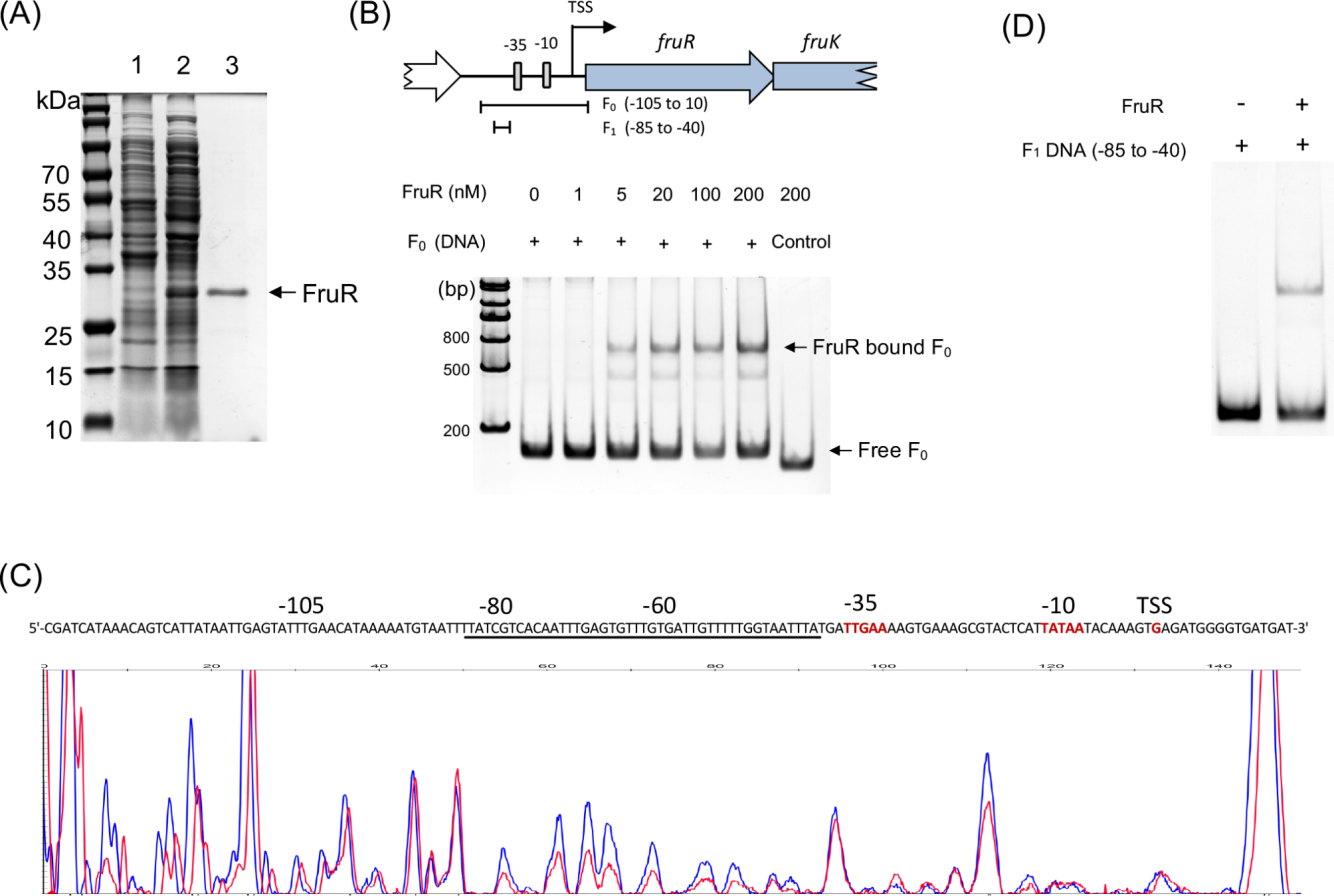
(**A**) Expression and purification of FruR protein. SDS-PAGE of cell lysates from *E. coli* BL21 (lane 1), BL21 carrying pET-22b (+)_*fruR* induced by IPTG (lane 2), and purified FruR protein (lane 3). (**B**) EMSA of the specific binding of purified FruR protein to the *fruRKT* promoter region F_0_ (-105 to 10). (**C**) DNase I footprinting assay to determine FruR binding site. The FruR protein protected region showed a significantly reduced peak intensities (red) pattern than seen in compared with those of the control (blue). (**D**) EMSA of the specific binding of purified FruR protein to the shorten *fruRKT* promoter regions F_1_ (-85 to -50)

To establish the direct involvement of the FruR protein in the regulation of the *fruRKT* operon, we conducted electrophoretic mobility shift assays (EMSA) to determine the binding between FruR and *fruRKT* operon promoter. In order to achieve this objective, we conducted the purification of the FruR protein, which had been overexpressed in *E. coli*, with a C-terminal 6×His tag (Fig. 4A). A 115 bp DNA fragment (F_0_), corresponding to the − 105 to 10 of the TSS from the *fruRKT* operon promoter, was used as a probe in the EMSA assay (Fig. 4B). The obtained results demonstrated the specific binding of FruR to the probe, which resulted in the appearance of shifted bands in a FruR concentration-dependent manner (Fig. 4B).

To precisely determine the binding site of FruR to the *fruRKT* promoter, a PCR-generated DNA fragment corresponding to the − 135 to 15 region upstream of the TSS was obtained, with FAM-labeling at the 5’ end. Subsequently, analysis of the FruR binding site within this fragment was conducted using the DNase I footprinting assay. Notably, the DNase I digestion assay revealed significant protection of the − 85 to -40 region upstream of the TSS (Fig. 4C), indicating this region as the precise FruR protein binding site. To provide further confirmation, an additional EMSA was performed, wherein only the specific region F_1_ fragment (-85 to -40) was utilized as a probe (Fig. 4D). Cumulatively, these findings conclusively illustrated that the FruR protein exhibited binding affinity towards a proximal region adjacent to the − 35 motif within the promoter region of the *fruRKT* operon.

### Fructose enhance the transcription of ***fruRKT*** genes

**Fig. 5:**
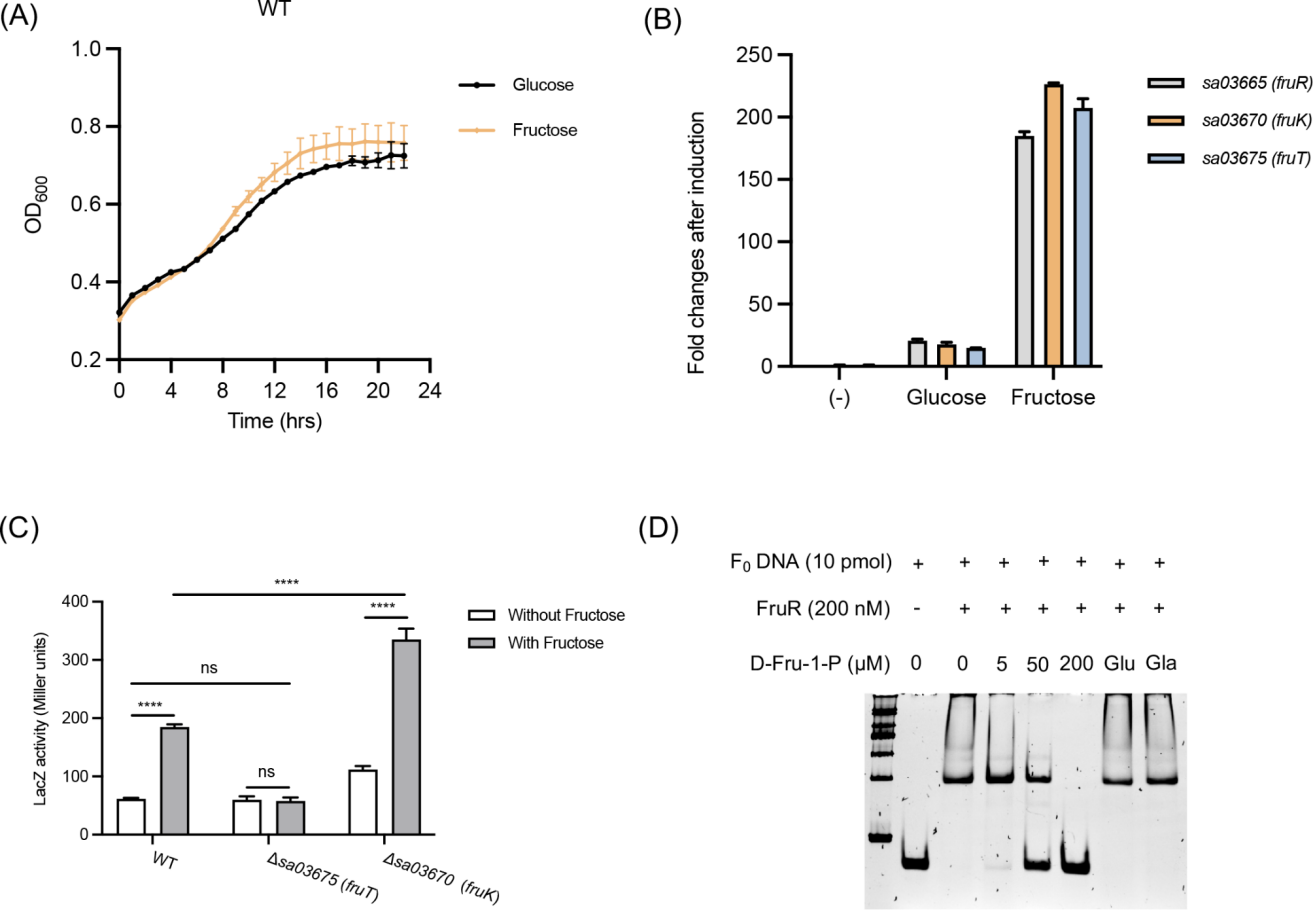
(**A**) Growth curves of *S. aureus* USA300 WT cultured in SMM minimal medium supplemented with 0.5% glucose or fructose. (**B**) RT-qPCR analysis of *fruRKT* gene expression levels in SMM minimal medium supplemented with 0.5% glucose or fructose. Expression levels were normalized to the baseline measurements obtained prior to the introduction of either sugar. (**C**) The pQLV003 derived P_*fruRKT*__*lacZ* reporter plasmid was transformed into *S. aureus* WT, Δ*03670* and Δ*03675* strains. The lacZ enzyme activity of the cell lysates of each strain growing in the presence or absence of fructose was measured. (^****^P ≤ 0.0001 relative to the level without fructose induction. ns, not significant). Each assay was performed three times and the data was presented as the mean plus standard deviation. (**D**) D-Fructose 1-phosphate (D-Fru-1-P) disrupted the binding of the FruR protein to the promoter sequence DNA fragment. Glucose (Glu) and galactose (Gla) at a concentration of 200 μM each were employed as controls

In order to investigate the carbon source-dependent expression of genes within *fruRKT* operon, the transcription levels were examined through RT-PCR experiments. For this purpose, total RNA was extracted from *S. aureus* USA300, which was cultivated in SMM minimal medium supplemented with glucose or fructose as a sole carbon source. Notably, both carbon sources supported *S. aureus* growth as evidenced by comparable growth patterns under each condition (Fig. 5A). Subsequently, RT-qPCR analysis was performed to determine the relative mRNA levels of the *fruRKT* genes in cultures grown in glucose- or fructose-containing medium, and compared to the baseline measurements obtained prior to the introduction of either sugar (Fig. 5B). The mRNA levels of *fruRKT* genes displayed a minor increase during growth in glucose-containing medium. Conversely, the *fruRKT* genes in fructose-containing medium exhibited significantly higher mRNA levels, which exhibited approximately 10-fold greater than those in glucose-containing medium (Fig. 5B). The obtained results distinctly demonstrated the transcriptional regulation of the *fruRKT* operon in response to fructose stimulation.

### Fructose-1-phosphate is the potential inducer that prompts the expression of ***fruRKT*** operon

To investigate whether fructose itself or an intracellular derivative of fructose acts as the inducer, we assessed the beta-galactosidase activity transcribed from the *fruRKT* operon promoter in different strains of *S. aureus* WT, Δ*03670* (*fruK*), and Δ*03675* (*fruT*) in the presence or absence of fructose as sole carbon source in the SMM medium. The results revealed that fructose induces the expression of the *fruRKT* operon in the WT strain, as indicated by significantly higher promoter activity in the presence of fructose compared to its absence (Fig. 5C). Interestingly, in the Δ*03675* (*fruT*) strain lacking the fructose-specific enzyme IIABC component of the PTS, responsible for the uptake and phosphorylation of fructose to fructose-1-phosphate, the promoter activity remained low and similar to the repressing condition observed in the WT when in the presence of fructose (Fig. 5C). This suggested that the inducer is not fructose itself but rather an intracellular derivative of fructose that processed through FruT. Intriguingly, the knockout of *sa03670* (*fruK*) gene, which encodes the 1-phosphofructokinase responsible for phosphorylating fructose-1-phosphate to fructose-1,6-bisphosphate, does not impair the induction of *fruRKT* operon expression by fructose (Fig. 5C). Notably, the expression level in the Δ*03670* (*fruK*) strain was even higher than that in the WT induced by fructose. It is postulated that the deletion of *sa03670* (*fruK*) gene reduces the consumption of fructose-1-phosphate, leading to its accumulation, thus suggesting that fructose-1-phosphate acts as the direct inducer of *fruRKT* operon expression. This hypothesis was further supported through the EMSA assay, demonstrating the disruptive effect of D-fructose-1-phosphate on FurR binding at the *fruRKT* promoter region. Consequently, this disruption mitigated the transcriptional repression exerted by FurR at the promoter site (Fig. 5D).

### Specificity of FruR regulation at the genome scale

**Fig. 6:**
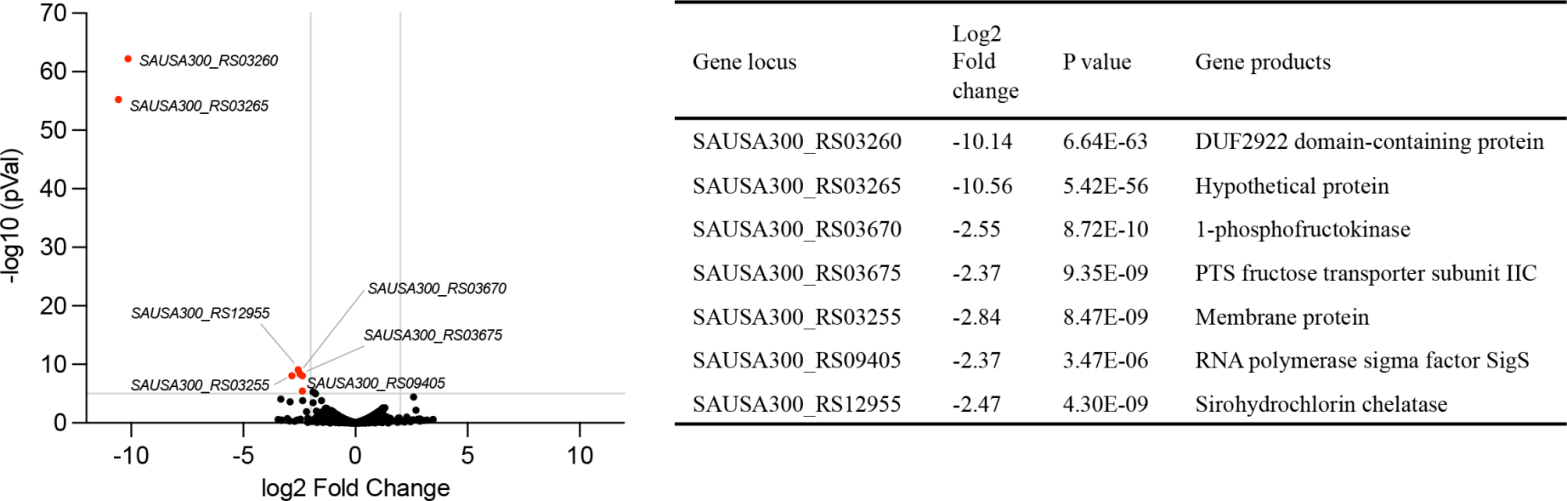
Differential gene expression plots in the *sa03665* (*fruR*) overexpression strain compared to the Δ*03665* mutant. The genes that differ significantly (log2 fold ≥ 2, -log10 *p*-value ≥ 5) were highlighted as red dots, and the corresponding products of these genes were listed

To analyze whether genes other than those of the *fruRKT* operon are regulated by FruR, the genome-wide expression profile of Δ*03665* (*fruR*) and an *fruR* gene overexpression strain Δ*03665*_com was determined by RNA-seq. Compared to the Δ*03665* (*fruR*) strain, the *sa03665* gene overexpression strain exhibited no up-regulated gene but few significant down-regulated genes, including the proved target genes *sa03670* (SAUSA300_RS03670) and *sa03675* (SAUSA300_RS03675) from the *fruRKT* operon (Fig. 6). Other than that, five genes encoding RNA polymerase sigma factor SigS, sirohydrochlorin chelatase, and function unknown protein were found down-regulated in the *fruR* overexpression strain (Fig. 6). The expression profile of each strain was stored in the BioProject (PRJNA990971) at NCBI.

## Discussion

Transcriptional regulators categorized under the DeoR family are widespread in bacteria and exhibit conserved domain architectures, which consistently comprise an N-terminal HTH DNA binding domain and a C-terminal sensor domain [8, 9]. Likewise, SA03665 also harbors these conserved domains, suggesting that the gene encoding SA03665 is likely a DeoR transcriptional regulator, as per gene annotation. DeoR proteins in bacteria exhibit diverse functionalities, involving sugar metabolism, morphological development, antibiotic production, and biofilm formation [3, 4, 6, 10]. Phylogenetically, SA03665 bears a strong resemblance to the DeoR protein in *S. citri*. In the latter, the DeoR coding gene is located upstream of a fructose permease gene and a phosphofructokinase gene, constituting a single operon [3]. It is worth noting that in *S. citri*, DeoR is responsible for regulating the expression of these two genes, which subsequently govern the uptake and catabolism of fructose [3]. Based on the analogous genomic context of DeoR in *S. aureus*, we proposed that SA03665 (FruR) has great potential in regulating the downstream fructose genes, hence facilitating fructose utilization in this bacterium. As anticipated, the involvement of *fruK* and *fruT* genes in fructose utilization was subsequently confirmed through their individual deletion, resulting in compromised growth of *S. aureus* under minimal medium conditions utilizing fructose as the sole carbon source (Fig. 2).

Fructose is predominantly metabolized via the phosphoketolase pathway in bacteria, which involves the uptake of fructose via membrane-spanning proteins such as fructose permease, simultaneous phosphorylation to fructose-1-phosphate, and subsequent phosphorylation by 1-phosphofructokinase (FruK) to generate fructose-1,6-bisphosphate. This is then processed through the citric acid cycle, generating the necessary energy for bacterial metabolism [1].

In *S. aureus*, *sa03670* (*fruT*) gene is predicted to encode the permease of the fructose phosphotransferase. Despite the fact that the deletion of the *fruT* gene has been demonstrated to impair the utilization efficiency of *S. aureus* towards fructose, the *fruT* mutant can still metabolize this sugar (Fig. 2B). These findings suggest that alternative pathways for both transport and catalysis of fructose to fructose-1-phosphate potentially exist within the *S. aureus*. However, deletion of the *sa03675* (*fruK*) gene, responsible for the 1-phosphofructokinase, completely eliminated the ability of *S. aureus* to utilize fructose, indicating that FruK encoded by *sa03675* gene is the potentially exclusive 1-phosphofructokinase in the pathway of fructose catabolism in *S. aureus*.

DeoR-type regulators are well-documented as repressors of sugar-specific PTS genes, that facilitate the absorption and metabolism of sugars in Gram-positive bacteria [5, 6, 11]. However, an unusual case is presented in *S. citri*, where the DeoR-type regulator FruR functions as a transcriptional activator of a fructose operon. Our research utilizing RT-qPCR analysis demonstrated a marked increase in *fruKT* gene expression in the *fruR* knockout mutant when compared to WT. Furthermore, the *fruRKT* promoter displayed greater activity in the *fruR* knockout mutant. These findings align with the general function of DeoR-type regulators as transcriptional repressors of nearby genes, observed in similar studies.

The majority of DeoR-type transcriptional repressors have been shown to inhibit gene expression of their neighboring genes by binding to their respective promoter regions, impeding the recognition of RNA polymerase and obstructing transcription initiation [4, 9, 12]. Currently, two sets of motifs have been identified as DeoR protein target binding sites. One motif was located within a range of + 23 to -59 nucleotides from the *fruR* start codon, while a second motif consisting of four adjacent repeats of 10 base pairs was observed at positions − 113 to -20 from the *fruR* start codon [5]. Notably, the second motif overlapped with the -35 box sequence of promoters where detection of promoter activity was inferred via the − 10 and − 35 consensus sequences.

As the two aforementioned motifs were absent from the promoter region of *fruR* in the *S. aureus* USA300 strain, we conducted an investigation into the binding site of FruR using a DNase I footprinting assay on a fragment amplified from − 135 to + 15 of the *fruR* TSS. Both EMSA and DNase I footprinting assays revealed that the binding site of FruR in *S. aureus* was located between − 85 and − 40 nucleotides from the transcription start site (Fig. 4). Although this binding site is not directly located within the − 35 to -10 RNA polymerase binding region, a distance of 10 base pairs from the − 35 motif could potentially be adequate for FruR-mediated transcriptional repression.

Considering that the transcription of the *fruRKT* operon was found to be enhanced under fructose stimulation, it becomes evident that there is a transcriptional regulation of the *fruRKT* operon affected by an inducer. This inducer could potentially be fructose itself or an intracellular derivative of fructose. To investigate this further, we examined the expression pattern of the *fruRKT* operon in Δ*03675* (*fruT*) and Δ*03670* (*fruK*) mutants under fructose induction.

In the main fructose utilization pathway, fructose enters the cell and is simultaneously phosphorylated to fructose-1-phosphate by a membrane-spanning protein FruT. Fructose-1-phosphate is then further phosphorylated to fructose-1,6-bisphosphate by ATP and FruK. In the Δ*03675* (*fruT*) mutant of *S. aureus*, the transport of fructose into the cell is impaired. Under this condition, the expression of the *fruRKT* operon in response to fructose is significantly lower compared to the WT induced by fructose. This suggests that the inducer responsible for the enhanced *fruRKT* operon expression is an intracellular fructose-derived molecule, specifically fructose-1-phosphate or fructose-1,6-bisphosphate [1].

Interestingly, when we examined the expression of the *fruRKT* operon in the Δ*03670* (*fruK*) mutant, we observed a significantly higher level of enhanced expression compared to the WT under fructose induction. The disruption of the *sa03670* (*fruK*) gene prevents the conversion of fructose-1-phosphate to fructose-1,6-bisphosphate, resulting in the accumulation of fructose-1-phosphate. The higher level of enhanced *fruRKT* operon expression in the Δ*03670* (*fruK*) mutant suggests that fructose-1-phosphate may act as a direct inducer of *fruRKT* operon expression. Since FruR acts as a transcriptional repressor of the *fruRKT* operon, it is possible that the induction of *fruRKT* operon by fructose-1-phosphate is achieved by inhibiting the activity of FruR. Fructose-1-phosphate may interact with FruR and disrupt its binding to the promoter region of the *fruRKT* operon, leading to increased expression.

The DeoR-type fructose operon transcriptional regulators have been reported to regulate the expression of the *fru* operon with high specificity in certain bacteria, such as *L. lactis* [5]. However, they have also been found to be involved in the regulation of other genes as seen in *C. glutamicum* [4]. In the case of FruR in *S. aureus*, besides regulating the genes from the *fruRKT* operon, only five other genes have been identified as target genes of FruR. However, upon analysis of the promoter regions of these genes, no similar binding sites of FruR to *fruRKT* operon were found. This suggests that the FruR regulator in *S. aureus* may have high specificity for the *fruRKT* operon, and any other genes regulated by FruR are likely to be indirectly influenced by its activity.

The fructose operon in *S. aureus* provides a metabolic advantage by efficiently utilizing fructose as a carbon source, fueling the bacterium’s energy production and growth. This pathway equips *S. aureus* to thrive in fructose-rich environments, enhancing its survival. Additionally, diverse metabolic pathways, including this operon, boost the adaptability of *S. aureus* to various conditions, ensuring competitiveness across ecological niches. Understanding this operon sheds light on the metabolic strategies of *S. aureus*, nutritional preferences, and overall fitness in different environments. Moreover, unraveling the role of this operon is crucial in identifying potential antimicrobial targets and gene regulatory mechanisms within *S. aureus*, offering insights into its pathogenicity and interactions with hosts. This knowledge opens avenues for therapeutic interventions and disease management.

In summary, we have discovered a sugar phosphotransferase system (PTS) involved in fructose metabolism in *S. aureus*. This system consists of three genes, *fruRKT*, which are organized in an operon and transcribed from a single promoter located upstream of the first gene *fruR*. The expression of the *fruRKT* operon is regulated by a DeoR-type regulator encoded by the *fruR* gene. In the absence of fructose, FruR represses the expression of the *fruRKT* operon by binding to its promoter region. However, in the presence of fructose, the expression of the *fruRKT* operon is induced, potentially through the action of fructose-1-phosphate as a direct inducer. This PTS system, which we have identified, represents the first system involved in fructose metabolism that has been described in *S. aureus*.

## Conclusions

This study provides valuable insights into the significance of the fruRKT operon in the metabolism of fructose in *S. aureus*. The *fruK* and *fruT* genes, which are part of this operon, are demonstrated to play a critical role in the utilization of fructose. Furthermore, the research uncovers the regulatory role of FruR, a transcriptional regulator belonging to the DeoR family, as a repressor of the *fruRKT* operon. By identifying the specific binding site of FruR in the promoter region of the operon, the study elucidates the mechanism behind its regulatory function. Moreover, the study highlights fructose as an inducer of the *fruRKT* operon, likely through the involvement of fructose-1-phosphate. These significant findings enhance our understanding of fructose metabolism in *S. aureus* and offer potential avenues for targeted interventions against this pathogenic bacterium.

## Methods

### Bacterial strains, plasmids, and growth conditions

The *S. aureus* USA300_FPR3757 strain was used to generate all the *S. aureus* mutants. Plasmids used for *S. aureus* USA300 transformation were modified by *S. aureus* RN4220. All *S. aureus* transformants were obtained through electroporation as described previously [13]. *S. aureus* strains were cultured in tryptic soy broth (TSB) liquid medium at 220 rpm with agitation or on TSB agar (TSA) plates at 37 °C. *E. coli* strain DH5α was used for plasmid cloning and was grown in Luria-Bertani (LB) broth at 37 °C. The *E. coli* BL21(DE3) was used for protein overexpression. The pBT2 vector was implemented for the construction of the knockout plasmid [14]. Plasmid pQLV1025 was employed for the complementation of the knockout gene in the deletion mutant [15]. To determine the promoter activity, the promoter probe vector pQLV1003 [15], which harbors a *lacZ* reporter gene, was utilized in the beta-galactosidase assay. Antibiotics were included at the following concentrations where specified: 100 μg/mL ampicillin, 10 μg/mL tetracycline, and 25 μg/mL chloramphenicol.

### In silico analysis

The co-expression of *fruRKT* genes was predicted using the online analysis tool STRING (https://cn.string-db.org/). The sequence alignment between SA03665 (FruR) and characterized DeoR transcription factors from *Spiroplasma citri*, *Lactococcus lactis*, *Streptococcus gordonii*, *Shigella flexneri*, *Escherichia coli*, *Pseudomonas syringae*, *Bacillus subtilis*, *Streptococcus mutans*, and *Streptococcus pneumoniae* was performed by using ClustalW. The resulting phylogenetic tree was generated using the neighbor-joining method and the Jukes-Cantor protein distance model from the multiple alignments, as implemented in CLC Main Workbench (Qiagen).

### Determination of transcription site

To identify the transcription start site of the *fruRKT* operon, 5’-RACE was performed using the HiScript-TS 5’ RACE Kit (Vazyme, RA101) following its protocol. Briefly, total RNA was extracted and reverse-transcribed into cDNA by using the 5’ random primer provided by the kit. In this process, an adaptor sequence was ligated at the 5’ end of the first-strand cDNA. Subsequently, a 5’ TS oligo with the reverse complementary sequence of the adaptor at its 3’ end was used to amplified the second strand cDNA. After that, the 5’ TS oligo along with the primer QL0961 (R1), QL0962 (R2), or QL0963 (R3) (Supplemental Table 1), which are complementary to *fruR*, *fruK*, and *fruT* respectively, were used to amplified their 5’ end sequence. Following purification of the PCR products, sequencing was undertaken by using QL0961, QL0962, or QL0963 to obtain the transcription start site of *fruR*, *fruK*, and *fruT* genes.

### Construction of ***S. aureus*** gene deletion and complementary mutants

Gene deletion mutants, including those with *fruR* (Δ*sa03665*), *fruK* (Δ*sa03670*), *fruT* (Δ*sa03675*), or *fruKT* (Δ*sa03670_03675*) knockout, were successfully constructed by means of homologous recombination, following the described methods [16]. The corresponding complementary strains of each knockout mutant were obtained by expressing the deleted gene under the control of a constitutive promoter using a replicative vector, namely pQLV1025 [15]. All the primers employed in the generation of these mutants were described and listed in the supplemental Table 1.

### Evaluation of fructose utilization by different strains

Utilization of fructose by different strains was evaluated in a synthetic minimal medium (SMM) [17] supplemented fructose as sole carbon source. Overnight culture of each strain was prepared in the TSB medium, and subsequently bacterial cells were harvested and washed with PBS three times before being resuspended in the SMM medium supplemented with 0.5% fructose. 100 μL of bacterial suspension was pipetted into the wells in a 96-well plate, and the OD_600_ values were monitored using a Synergy H1 plate reader (Bio-Tek).

### RNA isolation, RT-qPCR, and RNA-seq

RNA isolation and RT-qPCR were conducted as previously described [16]. To analyze the expression levels of the *fruRKT* genes in SMM minimal medium supplemented with 0.5% glucose or fructose, bacterial cells in mid-exponential phase cultured in TSB medium were harvested by centrifugation and washed thrice with PBS. The remaining cells were resuspended in SMM minimal medium to OD_600_ = 0.2 and supplemented with 0.5% glucose or fructose, followed by 12 h of culture before RNA isolation and RT-qPCR analysis. Transcription levels of the *fruRKT* genes in SMM media with glucose or fructose were compared to their respective baseline transcription levels. The expression level of *16 S rRNA* gene was used as an internal control. The extracted RNA was utilized as a template control in RT-qPCR to mitigate potential genomic contamination. Primer details used in RT-qPCR are provided in Supplemental Table 1. Each assay was conducted using three biological replicates.

For the RNA-seq analysis aimed at elucidating the target gene of FruR, bacterial cells in the mid-exponential phase belonging to two strains, Δ*03665* (*fruR*) and Δ*03665*_com (overexpressing *fruR* gene), were harvested by centrifugation after culturing in TSB medium. The RNA extraction was carried out using an RNApure Bacteria Kit (CwBIO, Jiangsu, China) as per the manufacturer’s instructions. To remove rRNA, a Ribo-Zero rRNA Removal Kit (Gram-positive Bacteria, Illumina) was employed. Subsequently, cDNA library preparation and sequencing were performed by Personalbio Co. (Shanghai, China).

### Beta-galactosidase-based promoter activity assay

The activity of *fruRKT* operon promoter was evaluated by beta-galactosidase assay as described in our previous study [15]. The promoter sequence (P_*fruRKT*_) was amplified by PCR using primers QL1013 and QL1014. The generated fragment was subsequently cloned into pQLV1003 and transformed into *S. aureus* USA300 WT or Δ*03665* (*fruR*) mutant. Bacterial cultures of *S. aureus* USA300 WT or Δ*03665* (*fruR*) mutant containing the P_*fruRKT*__*lacZ* reporter plasmid was applied to TSA plate containing the X-gal with the concentration of 25 μg/mL. The plate was then incubated at 37 °C, and the color of the colony was diligently monitored and photographed after 48 h. Additionally, the enzyme activity of the cell lysates of these strains was quantified in accordance with previously described methods [15]. To assess the LacZ activity of various mutants harboring the P_*fruRKT*__*lacZ* plasmid, the bacterial strains were cultivated in SMM medium supplemented with 2% fructose as the sole carbon source for a duration of 12 h. Subsequently, the cellular lysates were subjected to enzyme activity quantification.

### Expression and purification of FruR protein

The *fruR* gene was codon optimized for expression in *E. coli*, and was synthesized for cloning into pET-22b (+) between NdeI and XhoI sites. *E. coli* BL21 (DE3) was transformed by the resulting plasmid to generate the expression strain. For production, a 1:50 dilution of an overnight preculture of the overexpression strain was grown in 500 ml of LB supplemented with 100 μg/mL ampicillin. After reaching OD_600_ 0.8 at 37 °C, 0.5 mM IPTG was added to induce the production of His-tagged FruR over a 4-hour period at 30 °C. The cells were harvested by centrifugation at 5000 g for 15 min at 4 °C and washed in PBS. Bacteria were lysed through incubation for 30 min at 37 °C with 0.1 mg/ml lysozyme in the same buffer supplemented with 1 mM PMSF, followed by 1 min of sonication to complete lysis. The soluble fraction was recovered via centrifugation at 12 000 g for 30 min at 4 °C and filtered through a Ni^2+^-NTA agarose column equilibrated with lysis buffer. The column was thoroughly washed in wash buffers A and B (PBS; PBS with 500 mM NaCl and 20 mM imidazole). FruR_6×His-tagged was then eluted via an increasing imidazole gradient (50 mM to 400 mM). Protein-containing fractions were collected and analyzed by SDS-PAGE.

### EMSA assay

DNA fragment F_0_ was amplified by PCR using primers QL1003 and QL1004. The shorter fragment (F1) containing the target binding site was generated by annealing two reverse and complementary primers QL1449 and QL1450. Subsequently, approximately 10 pmol of the DNA fragments were incubated with purified FruR protein at 37 °C for 20 min in a binding buffer consisting of 10 mM Tris-HCl (pH 8.0), 50 mM NaCl, 1 mM EDTA, 1 mM DTT, and 5% glycerol. Various concentrations of FruR protein ranging from 1 to 200 nM were added to a total reaction volume of 20 μL. The samples were subjected to 5% native acrylamide gel electrophoresis in 0.5×TAE buffer. Subsequently, the gel was stained with GelRed and visualized using the ChemiDoc system (Bio-Rad). A 100 bp fragment downstream of the start codon of *fruR* gene was synthesized as a DNA control. In the EMSA assay examining the disturbance caused by D-Fructose 1-phosphate disodium (D-Fru-1-P) (MedChemExpress), the FruR protein, F_0_ fragment DNA, and varying concentrations of D-Fru-1-P (5, 50, or 200 μM) were co-incubated at 37 °C for 20 min.

### DNase I foot printing assay

A 150-bp fragment of the *fruRKT* promoter region was PCR-amplified using FAM-labeled primer QL1093 and QL1004. Eighty nanograms of the FAM-labeled promoter was then incubated with 500 nM FruR protein in a total volume of 20 μL binding buffer containing 10 mM Tris-HCl (pH 8.0), 50 mM NaCl, 1 mM DTT, and 5% glycerol. Promoter DNA without FruR protein was included as the control. Following incubation at 37 °C for 20 min, 0.1 U of DNase I (Thermo, EN0521) was added to the 20-μl reaction, which was then further incubated for 5 min at room temperature. The reaction was halted by heating at 65 °C for 10 min in the presence of 250 mM EDTA. The resulting DNA fragments were analyzed via capillary electrophoresis platform (ABI, 3730XL).

### Electronic supplementary material

Below is the link to the electronic supplementary material.

**Supplementary Material 1: Supplementary Table 1.** Primers used in this study

**Supplementary Material 2: Supplementary Information file 1.** The section depicted in Fig. 4A was extracted from this complete gel image, as indicated by the red box. **Supplementary Information file 2.** The section depicted in Fig. 4B was extracted from this complete gel image, as indicated by the red box. **Supplementary Information file 3.** The section depicted in Fig. 4D was extracted from this complete gel image, as indicated by the red box. **Supplementary Information file 4.** The section depicted in Fig. 5D was extracted from this complete gel image, as indicated by the red box.

## Data Availability

The dataset supporting the conclusions of this article is included within the article and its supplementary files. Transcriptome data of Δ*03665* (*fruR*) and *sa03665* gene overexpression strain was stored in the BioProject (PRJNA990971) at NCBI. Primers used in this study were listed in Supplementary Table 1.

## Abbreviations

5’-RACE: 5’ rapid amplification of cDNA end
EMSA: Electrophoretic mobility shift assays
PTS: Phosphotransferase system
SMM: Synthetic minimal medium
TSS: Transcriptional start site
