# Supplementary material for: Unveiling the fructose metabolism system in Staphylococcus aureus: insights into the regulatory role of FruR and the FruRKT operon in bacterial fitness
Source: BMC Microbiol. 2024 Jan 4;24:13. doi: 10.1186/s12866-023-03151-x (PMC10765703; doi:10.1186/s12866-023-03151-x)
Supplement: Supplementary file 2 — Supplementary Material 2: Supplementary Information file 1. The section depicted in Fig. 4A was extracted from this complete gel image, as indicated by the red box. Supplementary Information file 2. The section depicted in Fig. 4B was extracted from this complete gel image, as indicated by the red box. Supplementary Information file 3. The section depicted in Fig. 4D was extracted from this complete gel image, as indicated by the red box. Supplementary Information file 4. The section depicted in Fig. 5D was extracted from this complete gel image, as indicated by the red box. [file 12866_2023_3151_MOESM2_ESM.pdf]

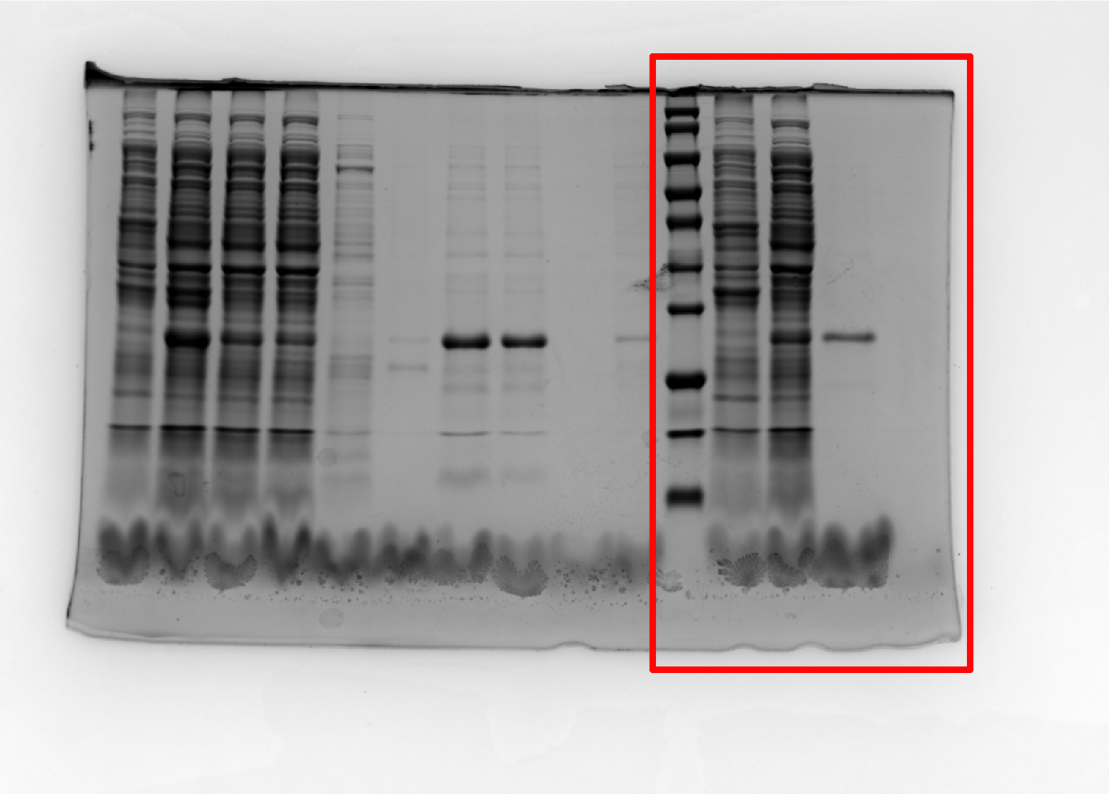

**Supplementary Information file 1.** The section depicted in Fig. 4A was extracted from this complete gel image, as indicated by the red box.

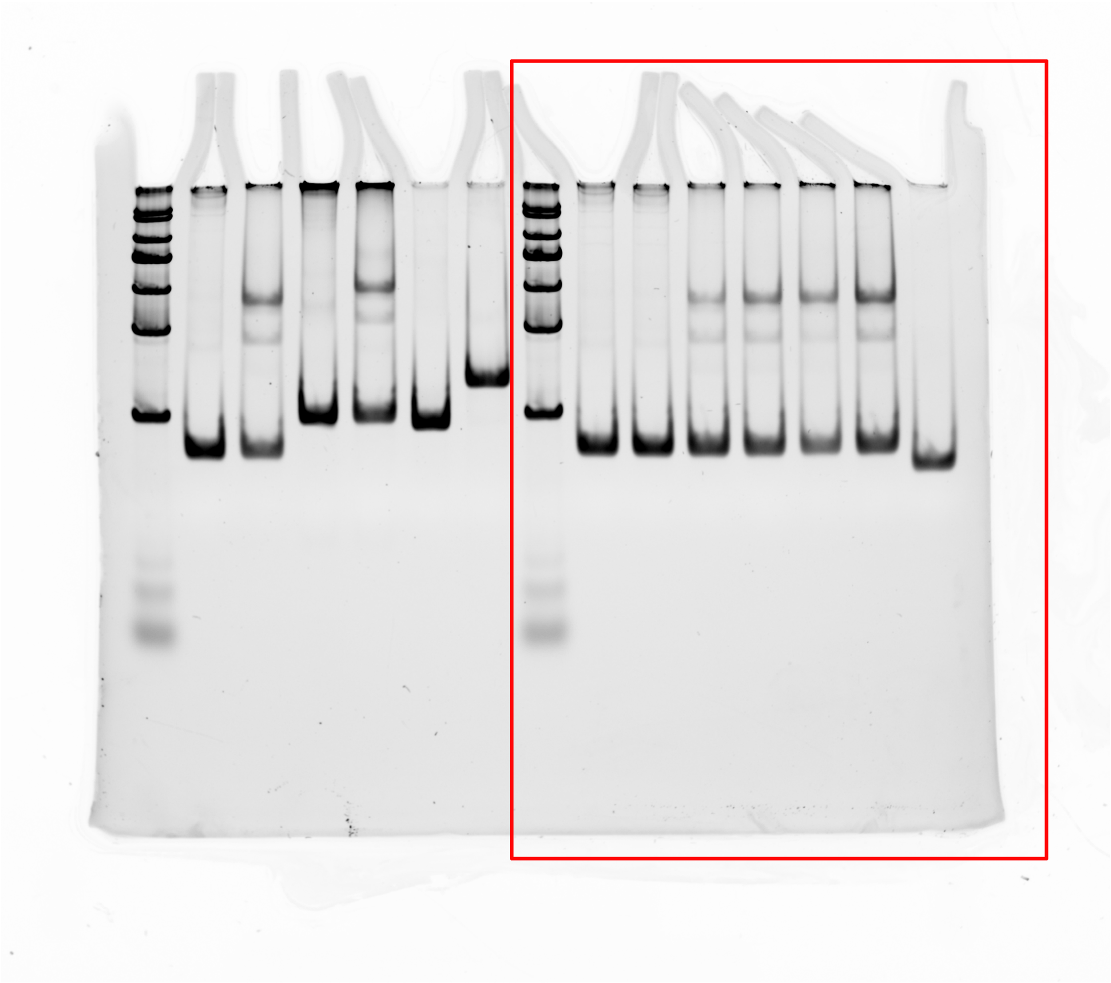

**Supplementary Information file 2.** The section depicted in Fig. 4B was extracted from this complete gel image, as indicated by the red box.

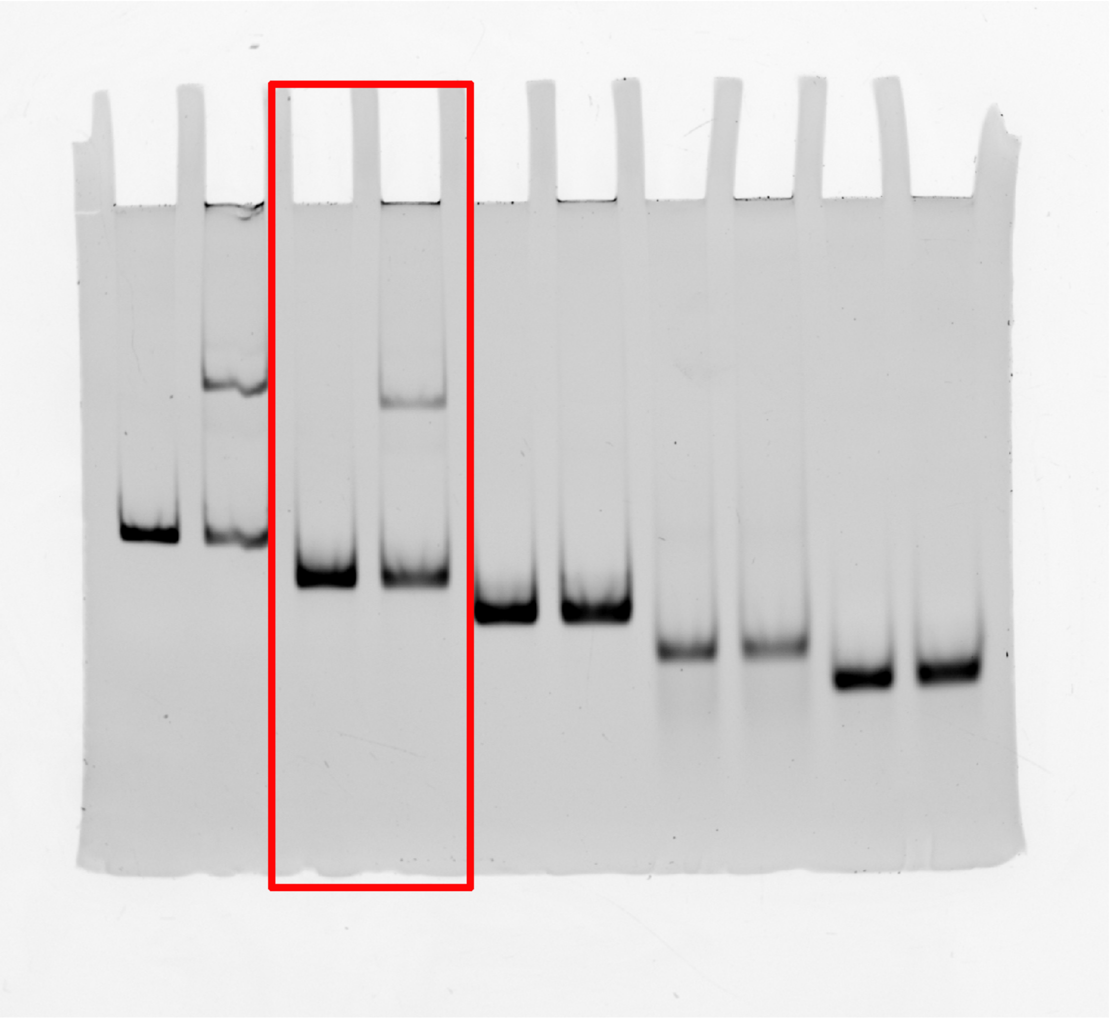

**Supplementary Information file 3.** The section depicted in Fig. 4D was extracted from this complete gel image, as indicated by the red box.

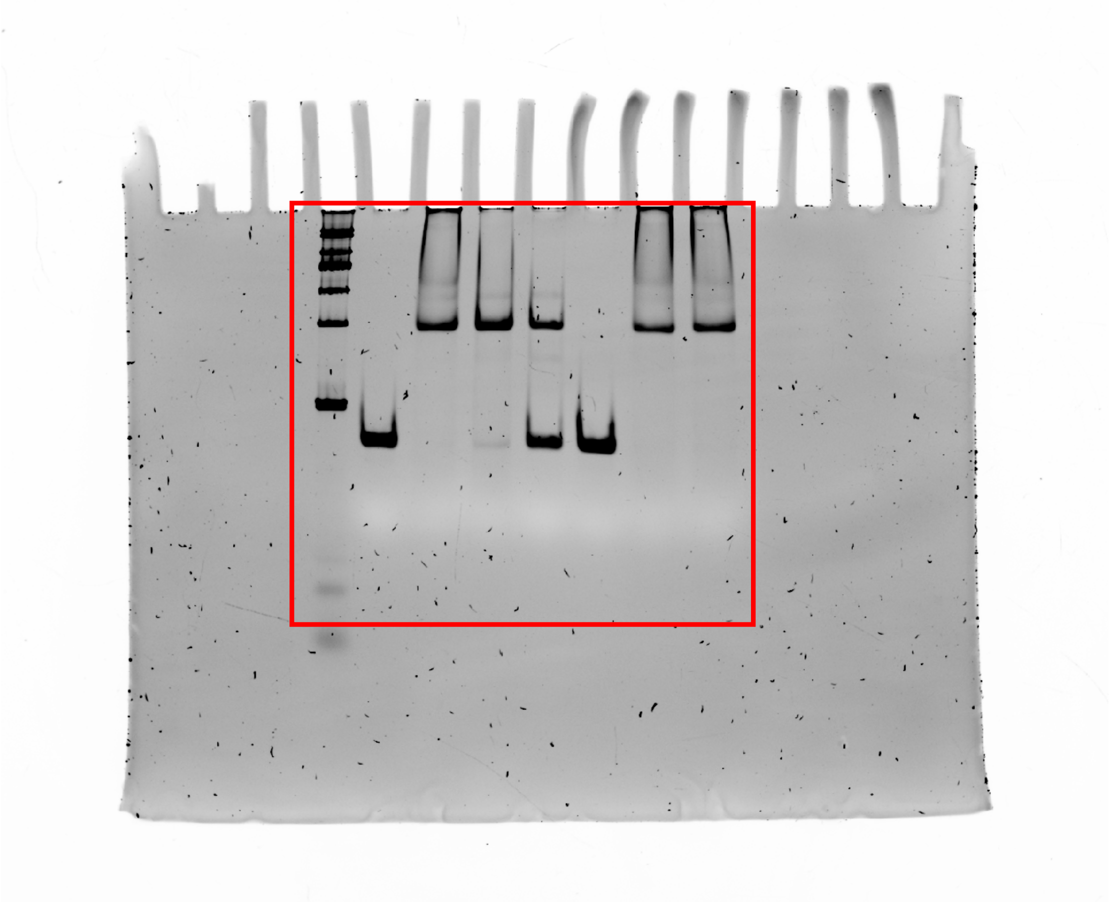

**Supplementary Information file 4.** The section depicted in Fig. 5D was extracted from this complete gel image, as indicated by the red box.
